# World Trauma Congress: when dreams come true

**DOI:** 10.1186/1749-7922-7-S1-S1

**Published:** 2012-08-22

**Authors:** Raul Coimbra, Gustavo Pereira Fraga, Sizenando Vieira Starling

**Affiliations:** 1 University of California, San Diego, USA; 2 University of Campinas, Campinas - SP, Brazil; 3 Hospital Joao XXIII, Belo Horizonte - MG, Brazil

## 

Trauma is a major public health problem worldwide. More than 5 million people die every year as a consequence of traumatic injuries. This disease does not distinguish between developed or underdeveloped countries; it is a major challenge to modern societies.

In many instances, injuries occur due to their responsible actions such as drug abuse, drinking and driving, etc. Interpersonal violence, suicides, and motor vehicle crashes, just to name a few of the more prevalent mechanisms of injury, require aggressive prevention strategies.

Social and economic inequalities leading to hunger, lack of access to healthcare, limited education, and violence are common in many underdeveloped countries. In those locations, the impact of injury is even greater and trauma care is sometimes neglected or inexistent. Trauma systems and adequate trauma care led by trauma / critical care / acute care surgeons are needed to fight this epidemic disease. The involvement of other health care groups: nursing, technicians, physical and occupational therapy, nutritional specialists, paramedics, emergency medical technicians, social workers, and medical specialists are paramount to provide comprehensive care to the injured patient.

Progress has been made recently in many parts of the world: systems are being developed, injury prevention and trauma care is being thought in many medical schools worldwide, professional organizations focusing on trauma care have grown in number, and some governments have been proactive in supporting and developing trauma systems.

Bringing all professionals involved in trauma care together for a few days of productive discussions, education, collaboration and networking in Rio de Janeiro, Brazil, is a dream coming true.

Why have we decided to organize the World Trauma Congress (WTC)? First and foremost, because the concept of trauma as a disease must be disseminated globally. It deserves the same attention and investment in care, research, and prevention, as any other disease. The WTC will bring together different nations, professional organizations, health care professionals, and students to learn, discuss, debate, advance knowledge, and hopefully increase awareness about this devastating disease.

Why have the WTC in Brazil under the auspices of the Brazilian Trauma Society (SBAIT)? In recent years, Brazil has experienced a marked economic growth and stability, despite the fact that many of the chronic social problems and inequalities still persist. The Brazilian government, aware of the crisis in emergency care and the importance of injury as a public health problem, took the lead and has proposed an ambitious plan to improve care in emergency departments by financing the development of a network of public hospitals which will care for medical emergencies as well as trauma. In addition, the pre-hospital system in Brazil is very well organized and widespread. However, many gaps still exist. There are no developed trauma systems in the country and hospitals caring for injured patients have no data collection tools (trauma registries) to measure their outcomes and to develop performance improvement initiatives. Furthermore, public hospitals are not always well equipped, care teams, in general, are not adequately trained beyond ATLS, post-injury rehabilitation in the public system is scarce, and injury prevention programs are almost nonexistent. SBAIT has been in the forefront of trauma education and is the representative of all trauma surgeons in the country.

Why organize the WTC in Rio de Janeiro? This marvelous city under the protection of Christ the Redeemer (“CORCOVADO”) with his arms open 24 hours a day (just like on call trauma surgeons) is one of the symbols of this country. The city of Rio de Janeiro will be one of the sites of the 2014 Soccer World Cup and will host the Summer Olympic Games in 2016. It just made sense to organize the largest trauma event in the world in Rio.

How was the idea of the WTC born? The idea was born immediately after the unforgettable Pan-American Trauma Congress in Campinas, Brazil in 2008, but it became solidified during the Annual Meeting of the largest surgical professional organization in the world, the American College of Surgeons, in 2009. Coincidentally, during that meeting, Drs. Coimbra and Fraga became aware that Rio de Janeiro had just been chosen to host the 2016 Summer Olympic Games. Immediately, we considered the possibility of gathering the trauma community and having the WTC in Rio. The first step was to gain support form professional trauma organizations worldwide. Although it may seem a very difficult task, all societies represented in the WTC immediately accept the idea. To all of them and their members we are forever grateful. The second step was to gather the support of Brazilian medical professional organizations, government, industry, interest groups, and universities. The response was overwhelmingly supportive as well. Most recently, the World Health Organization has provided its support to the WTC and will participate in the event.

An incredible number of people have been involved in the organization of the WTC. They have all worked very hard to make the WTC a memorable and unforgettable event. The WTC will be the largest trauma meeting ever organized in the world: 72 international speakers from 36 different countries, 150 Brazilian Speakers, more than 740 abstracts, 26 full manuscripts selected for publication in two scientific journals (The Journal of the Brazilian College of Surgeons and the World Journal of Emergency Surgery), and representation of more than 30 international trauma societies. During four days, more than three thousand participants will have a unique opportunity to exchange information, discuss, and learn from the world leaders in trauma care.

We hope that all participants feel as excited as we are with this fantastic opportunity to develop a world coalition to advance trauma care using the WTC as its platform on a regular basis. The WTC is a clear example that dreams eventually come true.

## Competing interests

The authors declare that they have no competing interests.

